# Spermine: A Hemoglobin Modifier That Reduces Autoxidation and Regulates Oxygen Delivery

**DOI:** 10.3390/ijms27031197

**Published:** 2026-01-25

**Authors:** Peilin Shu, Zongtang Chu, Guoxing You, Weidan Li, Yuzhi Chen, Huiqin Jin, Hong Zhou, Ying Wang, Lian Zhao

**Affiliations:** 1 Academy of Military Medical Sciences, No. 27 Taiping Road, Haidian, Beijing 100850, China; yk2748764926@163.com (P.S.); m18613105106@163.com (Z.C.); bjguoxingyou@163.com (G.Y.); lwd_dan@163.com (W.L.); chenyuzhi_buct@163.com (Y.C.); jinhqjc@outlook.com (H.J.); zhouhtt1966@163.com (H.Z.); 2 Air Force Medical Center, Air Force Medical University, Beijing 100142, China

**Keywords:** hemoglobin-based oxygen carriers, hemoglobin oxygen affinity, P50 value, spermine, antioxidant activity

## Abstract

One of the major factors currently hindering the development of hemoglobin-based oxygen carriers (HBOCs) is the autoxidation of hemoglobin to inactive methemoglobin (MetHb). The effects of spermine on the stability, aggregation, structure, and function of adult hemoglobin (HbA) were studied. The interaction of spermine with HbA was elucidated by dynamic light scattering, colloid osmotic pressure measurements, thermal denaturation analysis, static light scattering, and oxygen dissociation assay. The antioxidant capacity of spermine was confirmed through UV–vis spectroscopic recordings, calculations of MetHb formation, and hydroxyl radical scavenging. The P50 value was determined by the oxygen dissociation curve to investigate the roles of spermine in increasing HbA’s oxygen affinity. The pH-dependent affinity between spermine and HbA was validated through surface plasmon resonance experiments. The transformation of HbA’s partial α-helix to a β-sheet structure induced by spermine was clarified using a microfluidic modulation spectrometer. The binding of spermine to βASP99, βGLU101, αTHR38, and αASN97 on HbA and the conformational shift in HbA towards the ‘R’ state were investigated via molecular docking and molecular dynamics simulations. In a word, spermine can enhance the oxygen affinity of HbA, effectively reduce autoxidation, and hold promise for applications in the research of HBOCs or hemoglobin modification.

## 1. Introduction

Blood transfusion is a life-saving intervention that involves standardized procedures [1]. Emergency blood transfusion plays a crucial role in saving lives during life-threatening events like accidents, natural disasters, battlefield injuries, or situations resulting in acute blood loss. It also benefits individuals with conditions such as cancer (e.g., leukemia or lymphoma), anemia, or bleeding disorders (e.g., hemophilia or von Willebrand disease) by improving their health outcomes. Moreover, blood transfusion is essential in various surgical procedures like organ transplantation, cardiovascular surgery, and major trauma surgery to compensate for blood loss during the surgery [2].

However, due to the blood shortage, storage damage, and concerns about transfusion-transmissible diseases such as AIDS, clinical blood transfusion is restricted. Thus, the development of hemoglobin-based oxygen carriers (HBOCs) has emerged as a pressing and significant option to ensure and fulfill the requirements of health. HBOCs, derived from human or animal hemoglobin (Hb), are obtained through processes such as polymerization, modification, or encapsulation [3]. As a prominent candidate area of research in the field of blood substitutes research, it has the potential to be utilized in treatments of hypoxic diseases, for example, hemorrhagic shock, and in the perfusion and preservation of transplanted organs [4,5,6,7].

Although it exhibits favorable characteristics in terms of oxygen binding and release, unmodified Hb can lead to significant adverse reactions [8]. It has been confirmed that in natural red blood cells, the transformation of Hb to methemoglobin (metHb) is prevented and reversed by a group of antioxidant enzymes and reducing agents. However, during preparation, HBOCs lose their redox environment. HBOC-201, which lacks “Cytochrome-b5 reductase” (NADH-dependent metHb reductase, EC 1.6.2.2) [9,10], generates free radicals like superoxide anion and hydrogen peroxide while supplying oxygen. This leads to the production of excess metHb, thereby reducing the capacity to release oxygen and consequently diminishing the amount delivered to tissues. Iron ions further mediate oxidative damage to endothelial cells, resulting in increased microvascular permeability [11,12]. Thus, it is crucial to consider measures to avoid the formation of metHb during the process of HBOCs development.

Minimizing HbA’s autoxidation while effectively delivering oxygen, neutralizing free radicals, and mitigating oxidative damage represents a critical issue that must be resolved in the development of HBOCs. Chang et al. have reported the development of a strategy that utilizes superoxide dismutase (SOD) and catalase (CAT) extracted from erythrocytes to cross-link Hb [13,14]. Alagic et al. have likewise designed novel chemical reagents for cross-linking and coupling Hb with SOD, demonstrating SOD activity and a reduction in Hb’s oxygen synergy [15]. Furthermore, the literature has documented new approaches to bolster the antioxidant capacity of HBOCs [16,17,18]. Pyrroloquinoline quinone (PDA), one of the most common melanin-like pigments with unique antioxidant properties, may potentially address the issue of Hb autoxidation during HBOCs preparation. Baidukova et al. showed that Hb nanoparticles synthesized by co-precipitation with an inorganic template and coated with PDA contain twice the amount of functionally active Hb compared to uncoated counterparts [19,20]. Research conducted by Zhang Jun et al. has demonstrated that Dextran cross-linked hemoglobin (Dex-Hb) can effectively reduce the autoxidation rate of Bovine Hemoglobin (bHb), thereby enhancing the performance of HBOCs [21]. However, these aforementioned processes of molecular modification or cross-linking are relatively complex. The use of organic solvents with antioxidant properties during preparation could potentially compromise the autoxidation efficacy of HBOCs.

**Figure 1:**
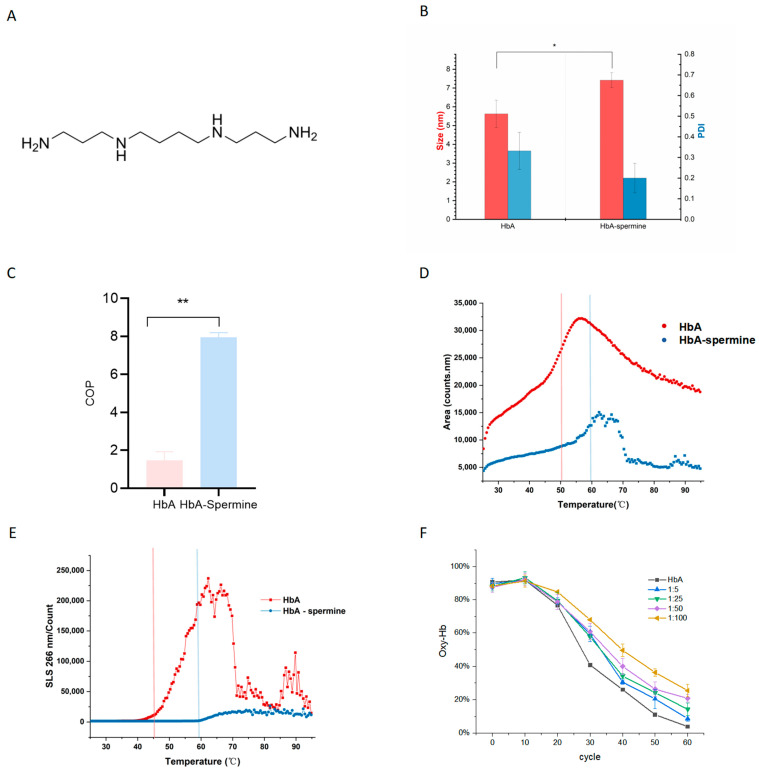
Characterization of HbA–spermine complex. (**A**) The chemical structure of spermine. (**B**) Size and PDI of HbA and HbA–spermine complex (1:50). (**C**) COP of HbA and HbA–spermine complex (1:50). (**D**) Thermal denaturation of HbA and HbA–spermine complex. The red line represents Tm1 of HbA, and the blue line represents Tm1 of HbA–spermine. (**E**) SLS during thermal denaturation of HbA and HbA–spermine. The red line represents Tagg266 of HbA, and the blue line represents Tagg266 of HbA–spermine. (**F**) Deoxygenated hemoglobin curve of HbA and HbA–spermine complex (1:25, 1:50, 1:100) over 50 cycles of deoxygenation. * indicates *p* < 0.05, ** indicates *p* < 0.01.

Spermine (Figure 1A) is a multimer present in all mammalian cells and serves various vital biological functions [22]. It plays a crucial role in regulating nucleic acid and protein structure, protein synthesis, protein–nucleic acid interactions, oxidative balance, and cell proliferation [23,24]. Additionally, spermine can act as a free radical scavenger or as a regulator of the Fenton reaction—a process that involves the oxidation of numerous organic compounds such as carboxylic acids, alcohols, and esters to inorganic states through a mixture of H_2_O_2_ and Fe^2+^ [25,26,27]. This property allows spermine to safeguard nucleic acids and cells against oxidative damage. GY11 fibroblasts, which are deficient in spermine synthesis, demonstrated a heightened sensitivity to hydrogen peroxide and an increased susceptibility to oxidative stress damage. This finding corroborates the antioxidant function of spermine [28,29]. Furthermore, as positively charged molecules, spermine has the ability to interact with a wide range of proteins, influencing their structure, function, interaction, and subcellular localization [30,31,32].

Due to its antioxidant activity and potential ability to bind to protein, spermine holds promise as an Hb modifier and a potential candidate molecule for preparing HBOCs. In this paper, we conduct a study to clarify the binding between spermine and Hb and characterize the structure, physicochemical properties, and functionality of the spermine–HbA compound.

## 2. Results

### 2.1. Spermine Forms a Complex with HbA

To demonstrate the formation of the HbA–spermine complex, the particle size, COP, Tm1, and Tagg of samples were analyzed. The hydrodynamic diameter of HbA increased significantly from 5.62 ± 0.74 nm to 7.42 ± 0.40 nm after incubating with spermine (Figure 1B) (*p* < 0.05, HbA–spermine complex vs. HbA). The PDI of HbA changed from 0.33 ± 0.90 to 0.2 ± 0.07 after incubation with spermine. Additionally, as illustrated in Figure 1C, the COP of HbA increased from 1.47 ± 0.46 to 7.97 ± 0.23 for the complex assembled using the 1:50 ratio (*p* < 0.01, HbA–spermine complex vs. HbA). The denaturation curve depicted a pronounced inflection point (Tm1) at 58.64 ± 0.62 °C in the figure. Upon incubating HbA with spermine at a molar ratio of 1:50, Tm1 was decreased to 50.10 ± 0.24 °C (Figure 1D) (*p* < 0.01, HbA–spermine complex vs. HbA). This suggests that the conformation of HbA altered upon spermine addition. Moreover, the aggregation temperature (Tagg266) of HbA increased from 45.15 ± 0.16 °C to 59.18 ± 0.67 °C upon spermine addition (Figure 1E) (*p* < 0.01, HbA–spermine complex vs. HbA). This suggests that the molecule was stabilized subsequent to the formation of the HbA–spermine complex [33]. As depicted in Figure 1F, samples attained peak oxygen saturation after 10 cycles of oxygenation. From 10 cycles to 60 cycles, a slower rate of deoxygenation was observed with increased spermine proportion. At the endpoint of deoxygenation, oxygen saturation decreased from 91.95% ± 1.25% to 3.87% ± 0.08% for HbA, from 91.67% ± 2.13% to 8.67% ± 1.69% for HbA–spermine (1:5), from 93.33% ± 3.67% to 14.24% ± 3.64% for HbA–spermine (1:25), from 92.16% ± 3.8% to 20.78% ± 2.32% for HbA–spermine (1:50), and from 91.30% ± 3.59% to 25.45% ± 3.86% for HbA–spermine (1:100). These findings collectively demonstrate that spermine effectively forms complexes with HbA.

### 2.2. Spermine Inhibits the Autoxidation of HbA

**Figure 2:**
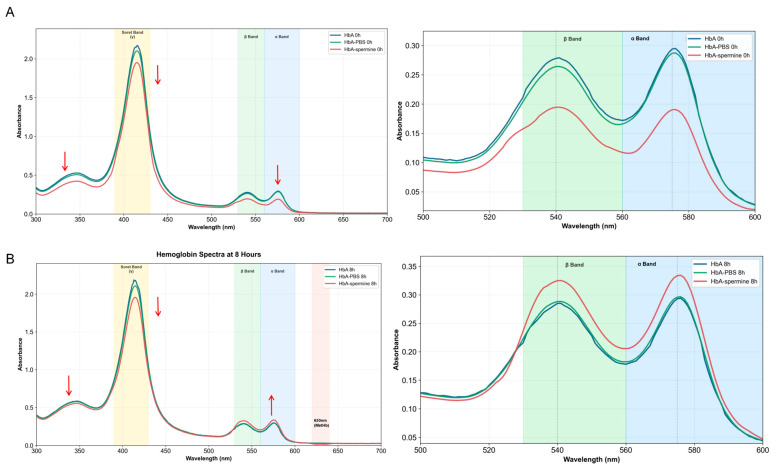
The UV-Vis spectra of HbA–spermine complex. (**A**) UV-Vis spectra of HbA and HbA–spermine complexes at 0 h. The Q band in the left figure is zoomed in as depicted in the right figure. At 0 h, spermine induces a hypochromic effect on the Q band of HbA. The red arrows indicate the changes in the globin band, S-band, and Q-band of hemoglobin upon the addition of spermine. (**B**) UV-Vis spectra of HbA and HbA–spermine complexes after 8 h. The Q band in the left figure is magnified as shown in the right figure. After 8 h, spermine causes a hyperchromic effect on the Q band of HbA. The red arrows indicate the changes in the globin band, S-band, and Q-band of hemoglobin upon the addition of spermine.

**Figure 3:**
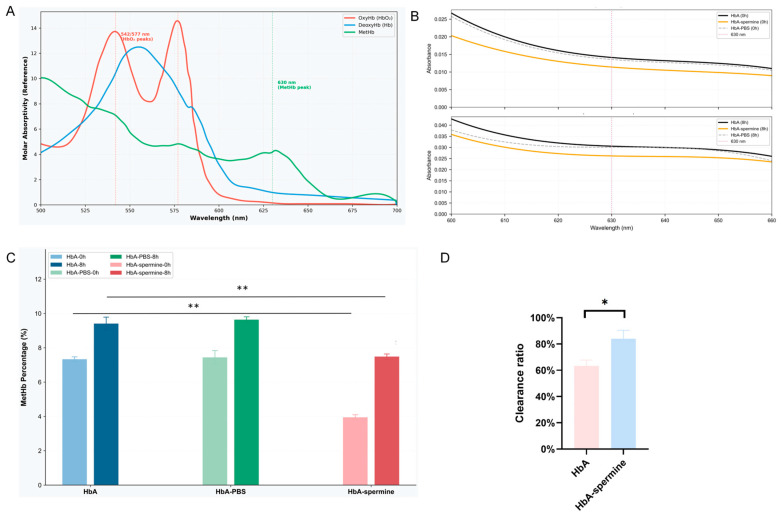
The antioxidant capacity of the HbA–spermine complex. (**A**) Basis spectra used to fit sample spectra for the determination of hemoglobin species concentrations. Basis spectra include oxyHb, deoxyHb, and MetHb. (**B**) Absorption spectra (630 nm) for HbA, HbA-PBS, and HbA-spermine at 0 h and 8 h. Compared to the HbA and HbA-PBS groups, the HbA–spermine complex exhibited a notably lower MetHb characteristic peak after 8 h of incubation. This observation indicates that spermine effectively inhibited MetHb formation, and this effect was not attributable to the PBS. (**C**) MetHb production of HbA, HbA-PBS, and HbA–spermine complexes. Spermine reduces the metHb formation of HbA. (**D**) Hydroxyl radical scavenging of HbA and HbA–spermine. Spermine enhances the scavenging of hydroxyl radicals in HbA. * indicates *p* < 0.05, ** indicates *p* < 0.01.

Spermine has antioxidant properties and can protect various biomolecules from oxidation [22]. Figure 2A shows the ultraviolet-visible (UV-Vis) spectra of HbA and HbA–spermine at 0 h at 37 °C. After spermine is added, the globin band (280 nm), Soret band (412 nm), and Q band (550–600 nm) of HbA all show a hypochromic effect, indicating that spermine affects the tertiary structure of HbA [34]. Figure 2B shows the UV-Vis spectra of HbA and the HbA–spermine complex after incubation at 37 °C for 8 h. The MetHb production of HbA and the HbA-spermine complex was calculated to verify antioxidant activity. Figure 3C illustrates the time-dependent increase in MetHb concentration. Starting from the 4th hour, MetHb concentrations of the HbA–spermine complex (1:50) were notably lower than those of HbA. The MetHb content in the HbA group was 9.4% ± 0.4% at the 8th hour, whereas it decreased to 7.5% ± 0.2% in the HbA–spermine group (*p* < 0.01, HbA–spermine complex vs. HbA). In addition, as depicted in Figure 3D, the hydroxyl radical scavenging efficiency of HbA was found to be 63.38% ± 4.42%, while that of the HbA–spermine complex reached 84.04% ± 6.42% (HbA–spermine complex, *p* < 0.01 vs. HbA). All the results proved the antioxidant capacity of the HbA–spermine complex.

### 2.3. Spermine Enhances the Oxygen Affinity of HbA, bHb, and Rat RBC

**Figure 4:**
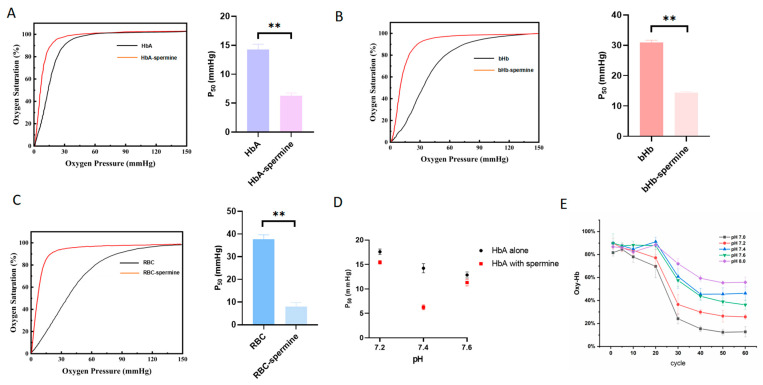
Effect of spermine on oxygen affinity of HbA, bHb, and RBC. (**A**) The ODC and P_50_ value of HbA and HbA–spermine (1:50). Spermine significantly decreased the P_50_ value of HbA. (*p* < 0.01). (**B**) The ODC and P_50_ value of bHb and bHb–spermine (1:50) (*p* < 0.01). Spermine significantly decreased the P_50_ values of bHb. (**C**) The ODC and P_50_ value of rat RBC and RBC–spermine (1:50). Spermine significantly decreased the P_50_ value of RBC. (*p* < 0.01). (**D**) Effects of spermine on P_50_ of HbA at pH 7.2, 7.4, and 7.6, among which spermine had the strongest regulatory effect on HbA at pH 7.4. (**E**) ODA curve of HbA alone and along with spermine at different pH levels over 50 cycles of deoxygenation. Error bars represent the standard deviation (SD). ** indicates *p* < 0.01.

After incubating with spermine, ODCs of HbA, bHb, and RBC shifted to the left (Figure 4A–C), indicating a significant decrease in the P_50_ value. The P_50_ value of HbA was 14.24 ± 0.94 mmHg and decreased to 6.25 ± 0.49 mmHg (1:50) with spermine incubation (*p* < 0.01, HbA–spermine complex vs. HbA). The P_50_ value of bHb was 30.87 ± 0.78 mmHg and significantly decreased to 14.43 ± 0.28 mmHg after spermine incubation (*p* < 0.01, bHb–spermine complex vs. bHb). Likewise, the P_50_ value of rat RBC was 37.79 ± 1.87 mmHg, which decreased to 8.04 ± 1.96 mmHg upon spermine incubation (*p* < 0.01, RBC–spermine complex vs. rat RBC). These results indicate a significant decrease in P_50_ for HbA–spermine, spermine–bHb, and spermine-RBC compared to HbA, bHb, and rat RBC, respectively.

Figure 4D depicts the impact of spermine on reducing the P_50_ of HbA at pH 7.2 (from 17.61 ± 0.57 mmHg to 15.44 ± 0.34 mmHg) (*p* < 0.05, HbA–spermine complex vs. HbA), pH 7.4 (from 14.24 ± 0.94 mmHg to 6.25 ± 0.49 mmHg) (*p* < 0.01, HbA–spermine complex vs. HbA), and pH 7.6 (from 12.86 ± 0.59 to 11.30 ± 0.66 mmHg) (*p* < 0.05, HbA–spermine complex vs. HbA). The reduction in P_50_ of HbA was most pronounced at pH 7.4. Figure 4E HbA–spermine showed that as the pH increased, the percentage of oxygenated hemoglobin exhibited a slower decrease. Additionally, the percentage of oxygenated hemoglobin displayed a relatively faster decrease at pH 7.4 compared to pH 7.6, indicating a slower oxygen release rate for the HbA–spermine at pH 7.4.

**Figure 5:**
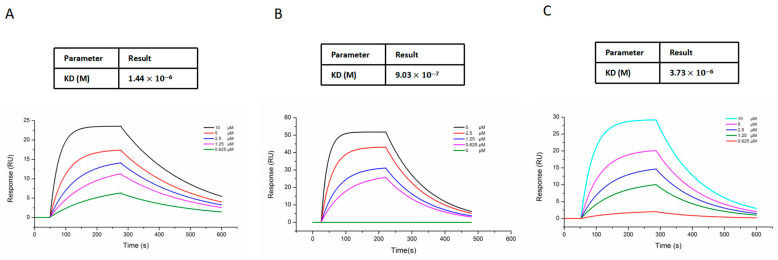
The impact of pH on spermine’s binding with HbA. (**A**) Spermine bound to HbA in a dose-dependent manner at pH 7.2 in the SPR assay. (**B**) Spermine bound to HbA in a dose-dependent manner at pH 7.4 in the SPR assay. The binding affinity of spermine to HbA is strongest at pH 7.4. (**C**) Spermine bound to HbA in a dose-dependent manner at pH 7.6 in the SPR assay.

To explore the P_50_ changes along with pH, the SPR assay was carried out. By monitoring the alteration in the SPR angle, kinetic parameters (KD, Kd, and Ka) were determined. The KD of spermine bound to HbA at pH 7.2 was 1.44 × 10^−6^, the KD of spermine bound to HbA at pH 7.4 was 9.03 × 10^−7^, and at pH 7.6 was 3.73 × 10^−6^ (Figure 5A–C). These results suggest a pH-dependent effect on the oxygen-carrying/releasing capacity of the HbA–spermine complex.

### 2.4. Spermine Has a Minimal Impact on the Secondary Structure of HbA

**Figure 6:**
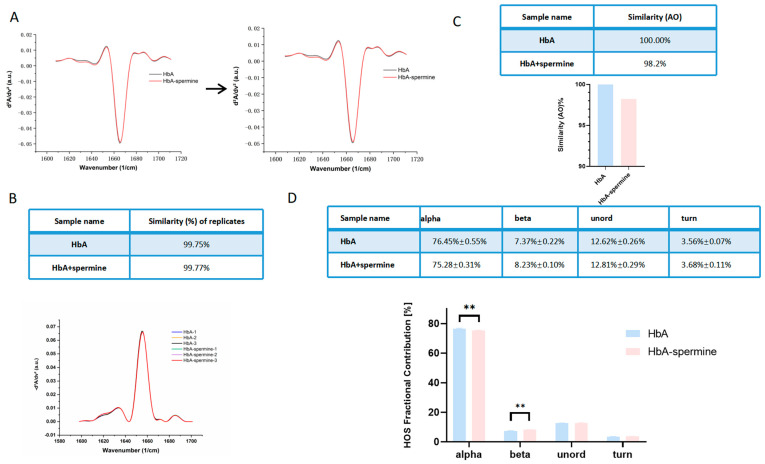
Secondary structure of HbA and HbA–spermine complex. (**A**) Absolute absorption spectra (left) are converted to second derivatives (right). (**B**) The similarity score of the three-time experiment was tested, with similarity values of 99.75% and 99.77%, respectively. These results indicate that the experimental system was stable and the findings are reliable. (**C**) The secondary structure of the HbA–spermine complex is slightly different from that of HbA. (**D**) Spermine has a slight influence on the HOS of HbA. Specifically, it leads to a reduction in the α helix structure of HbA, while promoting an increase in β sheet folding. ** indicates *p* < 0.01.

Figure 6A displays the absolute absorption spectra and second derivative spectra of HbA at a spectral range of 1701–1598 cm^−1^. 1652 cm^−1^ was the most intense, which indicates that HbA and HbA–spermine existed in an a-helix-rich conformation chiefly [35]. Figure 6B showed a similarity score of the three-time experiment was 99.75% for the HbA and 99.77% for the HbA–spermine (1:50), which indicates excellent system stability. Figure 6C showed that the similarity between HbA and the HbA–spermine complex is 98.2%. Figure 6D showed the results of the HOS histogram. Spermine decreased the alpha-helix content of HbA from 76.45% ± 0.55% to 75.28% ± 0.31% and increased the beta-sheet content of HbA from 7.37% ± 0.22% to 8.23% ± 0.10% (*p* < 0.01, HbA–spermine complex vs. HbA). In addition, spermine had little effect on random coil (from 12.62% ± 0.26% to 12.81% ± 0.29%) and beta-turns (from 3.56% ± 0.07% to 3.68% ± 0.11%) of HbA.

### 2.5. Spermine Binds to HbA at βASP99, βGLU101, αTHR38, and αASN97

**Figure 7:**
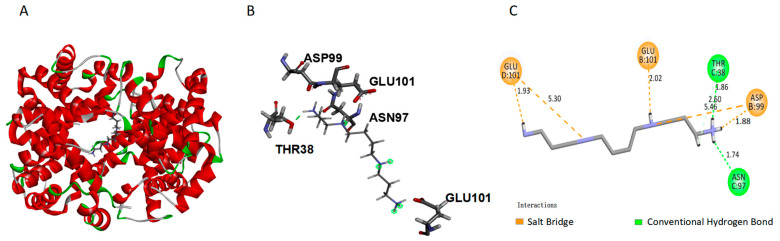
Specific binding pattern of spermine to HbA. (**A**) Binding pattern of spermine to HbA. Spermine is located in the central water cavity of HbA; HbA: red, cartoon pattern; spermine: silver gray, rod. (**B**) 3D image of spermine interaction with HbA. (**C**) 2D image of spermine interaction with HbA.

Molecular docking was used to predict the energetically favorable conformation of ligand molecules at active sites [36]. The docking conformation with the lowest free energy was selected for further analysis. Spermine can flexibly match and bind to the active pocket of HbA, and CDOCKER_ENERGY is 37.7944 kJ/mol. Further analysis revealed that spermine bound to the central hydrophobic cavity of HbA (Figure 7A). The interaction diagram between spermine and HbA is shown in Figure 7B,C, among which B represents a 3D image and C represents a 2D image. Figure 7C showed that spermine forms salt bridges (indicated by yellow dashed lines) with ASP99 in the β chain of HbA, as well as with GLU101 in the two β chains. Additionally, it forms hydrogen bonds (represented by green dashed lines) with THR38 and ASN97 in the α chain.

### 2.6. Spermine Stabilizes the Conformation of HbA in the “R” State

**Figure 8:**
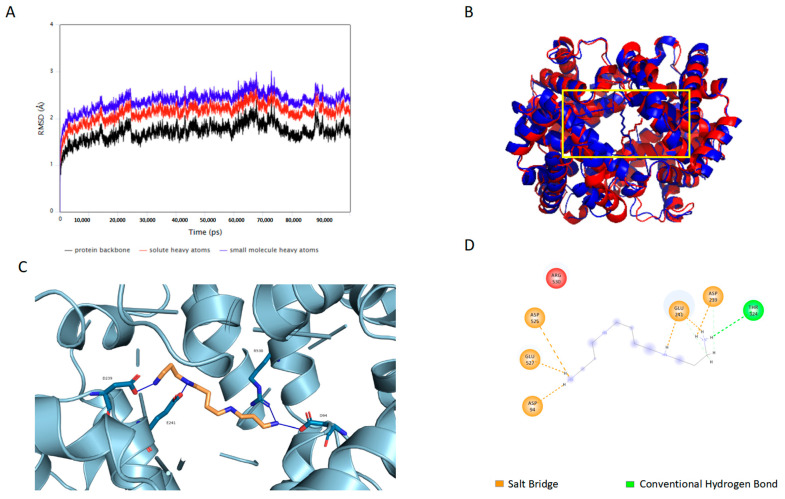
Conformational changes in the HbA–spermidine complex (**A**) RMSD of the interaction between spermine and HbA in MD of 100 ns. (**B**) The comparison diagram shows the conformation of the HbA–spermine complex before and after MD. The blue color represents the conformation of HbA, while the red color represents the conformation of HbA–spermine complex after simulation. (**C**) The conformation of the HbA–spermine complex, a 3D plot. (**D**) The interaction of HbA–spermine complex.

To assess the stability of spermine’s interaction with HbA, root mean square deviation (RMSD) values of the protein backbone in HbA and HbA–spermine complex were calculated. The RMSD plot of HbA and the HbA–spermine complex is illustrated in Figure 8A. In the dynamics simulation of 100 ns, trends of protein backbone, all solute heavy atoms, and small molecule heavy atoms are consistent. The RMSD value gradually increases and reaches stability at 10 ns, and the RMSD variation is within 2. In Figure 8B, the blue color represents the HbA structure, while the red color represents the HbA–spermine complex structure after the simulation. Following the formation of stable complexes, there is a significant size decrease in the β-cleft of HbA, which is primarily composed of β1H146 and β2H146. Figure 8C,D illustrate the complex. The results indicate that spermine interacts with HbA, forming salt bridges at Asp94, Asp239, Glu241, Glu525, and Asp527 of HbA, as well as hydrogen bonds at Thr324 of HbA. Table 1 displayed the binding energy via MM/GBSA calculations [37]. The results showed that the strongest component of the total binding free energy (−26.02 ± 10.09 Kcal/mol) is the ∆Gvdw (van der Waals forces). The ∆Gele (electrostatic attraction force) is relatively strong, but its effects are canceled out by the ∆Gpolar (polar solvent effect). ∆Ggas and ∆Gsolv energy also cancel each other out.

## 3. Discussion

Hb plays a crucial role in oxygen delivery. Despite the necessity to develop HBOCs due to the nephrotoxicity of free Hb, autoxidation presents a significant obstacle in product development [20,38,39]. Therefore, the development of HBOCs with free radical scavenging abilities holds great practical meaning.

Currently, there are various methods utilized for the development of HBOCs, including crosslinking, surface modification, and encapsulation [40,41,42]. However, adverse reactions such as oxidative damage originating from reactive oxygen species (ROS) arose during clinical trials [43]. In any case, it is essential for HBOCs to possess both oxygen-carrying/releasing capacity and antioxidant activity. Therefore, appropriate chemical modifications of Hb are necessary to reduce the MetHb content. One effective approach is to combine free radical scavengers with Hb, which can enhance the antioxidant capacity of HBOCs.

Spermine is a positively charged aliphatic molecule that exists in all cells [23,24]. It is abundant in living organisms and plays crucial biological roles, making it an essential component of life. Notably, spermine exhibits strong antioxidant capacity and can interact with various proteins, influencing their structure, function, and localization [30,32]. In in vitro studies, Yu CH et al. discovered that spermine could associate with histones, actin, and tubulin and pinpoint specific modification sites on these proteins. This interaction results in an increase in positive charges within the polyamine-integrated region, leading to a marked transformation in the protein’s structure [32]. Thus, we suggest that spermine has the potential to interact with HbA to make an antioxidant effect and verify the interaction. However, specific interactions between spermine and HbA, as well as its biophysical stability and functional changes, remain unknown. Therefore, this study investigates and verifies these aspects systematically.

We confirmed the formation of the HbA–spermine complex further. The size of the HbA–spermine complex revealed a significant increase in diameter from 5.62 ± 0.74 nm to 7.42 ± 0.40 nm, which provides the basis of evidence to further study the interaction between spermine and HbA. Additionally, the HbA–spermine complex displays the following increased COP, which is influenced by protein concentrations and molecular weights, from 1.47 ± 0.46 mmHg to 7.97 ± 0.23 mmHg. It is reported that the size of HBOC201, a kind of glutaraldehyde polymerized bHb, is 10.19 nm, and the COP of it is 17 mmHg [44]. The size of PDA-Hb is 8 nm [44]. The size of HBOCs influences their circulation time, biocompatibility, and potential side effects. And the optimal COP is crucial for maintaining water balance within and around blood vessels and blood volume. Upon the binding of spermine to HbA, COP approximates the optimal physiological state (the COP of plasma is approximately 25 mmHg). The decreased PDI in the HbA–spermine complex compared to HbA suggests that spermine does not promote Hb aggregation [2]. Further, the increased Tagg for the HbA–spermine complex also suggests that the protein is less likely to aggregate, thereby demonstrating that the two components form a systemically stable complex [33]. The conformation and stability of the HbA–spermine complex were subsequently analyzed. Upon binding with spermine, HbA exhibited a decrease in Tm1 and diminished thermal stability, suggesting a change in the conformation of HbA. The oxygen release of the HbA–spermine complex was proven to decrease with increasing spermine proportion. Fortunately, despite certain structural alterations, the HbA–spermine complex retains the oxygen-carrying/releasing capacity inherent to hemoglobin.

The antioxidant property is crucial for HBOCs’ development. The heme iron exists in a ferrous state (Fe^2+^), but it can undergo oxidation to the ferric state (Fe^3+^), impairing its ability to effectively carry or release oxygen. Spermine has the ability to protect various molecules from oxidation. For example, spermine effectively inhibits ROS-induced DNA damage, with the most significant inhibition observed at concentrations ranging from 1 to 2 mM [27]. Our data clarifies that spermine inhibits the production of MetHb, and this inhibitory effect is maintained over time. Furthermore, the rate at which hydroxyl radicals are scavenged was examined to evaluate the antioxidant potential of spermine. Hydrogen peroxide interacts with Fe^2+^ via the Fenton reaction and produces hydroxyl radicals. The results revealed that the hydroxyl radical scavenging of HbA alone did not exceed 10%. In contrast, the hydroxyl radical scavenging of HbA–spermine significantly increased to 63.38% ± 4.42%, indicating a potent antioxidant capacity. These findings are corroborated by the UV–vis spectroscopy experimental results, spermine making an effect on the Q band (550–600 nm) of HbA, and MetHb formation studies. The hyperchromic effect observed in the Q band of the HbA–spermine complex, compared to HbA alone, also indicates that spermine effectively inhibits MetHb production [45]. The antioxidant property of HBOCs is a significant factor in medical applications. The Hemolink, an o-raffinose cross-linked HBOC, has a MetHb content of 15.0%, which can potentially influence the oxygen-carrying/releasing capacity of HBOCs. The recognized free radical (ABTS•+) scavenging activity of PDA-Hb is 51%, and the MetHb is significantly reduced to 10.7% [2,21]. In this study, the metHb of HbA–spermine was 5.71 ± 9.0% after a 4 h period. And the hydroxyl radical clearance reached 63.38% ± 4.42%. Thus, comparatively, HbA–spermine demonstrated a significantly stronger antioxidant capacity.

Furthermore, a thorough examination was conducted to assess the impact of the interaction with spermine on HbA’s oxygen-carrying/releasing capacity, which shows highlighted alterations [34,45,46]. First, we performed ODC tracing and simultaneously evaluated the effect of spermine on the oxygen affinity of HbA, bHb, and rat RBC. The oxygen partial pressure corresponding to 50% oxygen saturation in the ODC is denoted as P50, which serves as a measure of the oxygen affinity. Following the binding of spermine, the ODC of HbA, bHb, and RBC all exhibited a noticeable left shift, resulting in lower P50 values and higher oxygen affinity. The enhanced oxygen affinity observed in HbA–spermine corroborates the findings from the mutual thermal stability assay. This heightened oxygen affinity is linked to a transition of HbA into an ‘R’ state, which induces a conformational alteration and results in a reduction in Tm1. The chemical modification of Hb generally yields HBOCs with an elevated oxygen affinity. This is evident in the products of PEGylated hemoglobin Sanguinate and Hemospan (MP4), which exhibit P50 values of 7–16 mmHg and 5–6 mmHg, respectively, similar to the HbA–spermine. These kinds of HBOCs could release oxygen solely to peripheral tissues and avoid the vascular activity effectively [47]. Moreover, the ODC maintained its S-shape, suggesting that HbA still maintains its synergistic effect. Furthermore, ODA and ODC collectively indicate that under pH 7.2, pH 7.4, and pH 7.6 conditions, spermine can reduce the P50 value of HbA universally. However, at pH 7.4, the HbA–spermine complex has the strongest P50 variation. To explain this phenomenon, the SPR assay with pH at 7.2, 7.4, and 7.6 was carried out. The value of KD (Kd/Ka) signifies the extent of dissociation of the receptor–ligand complex at equilibrium. A decrease in the KD value corresponds to an increase in affinity. It proved that the interaction between spermine and HbA was strongest at pH 7.4, while it was weakest at pH 7.6. The binding with spermine and oxygen affinity variation depending on pH value plays a crucial role in regulating oxygen uptake and release by HbA in the lungs and tissues. It means spermine could improve oxygen binding in lung tissue (pH 7.6, increased oxygen affinity), sustain stable oxygen transport (pH 7.4, more increased oxygen affinity), and did not interfere with the release of oxygen by HbA in tissues (pH 7.2, decreased oxygen affinity vs. pH 7.4). Overall, these findings about the oxygen-carrying/releasing capacity highlight the potential use of the HbA–spermine complex as a promising candidate for HBOCs.

Alterations in the Tm1 value and P50 suggest potential modifications to HbA structure. Besides the Q band, our results reveal that spermine also has effects on the globular band (280 nm) and the Soret band (412 nm) of HbA. The globular bands correspond to the absorbance of chromophore amino acid residues, such as Tyr and Trp, at 280 nm [48]. The electron transition within the porphyrin ring of the heme group displays a peak at 412 nm, known as the B band or Soret band [49]. The hyperchromism of the globin peak is associated with increased hydrophobicity around surface aromatic residues due to interaction with spermine, whereas the hypochromism of the Soret band relates to spermine penetration into the hydrophobic heme pocket, which increases local hydrophilicity by introducing water molecules [34]. The changes observed in the UV-Vis spectra reveal perturbations in the local tertiary structure, particularly within the microenvironment of the heme pocket. These findings suggest that spermine binds specifically to HbA, inducing local conformational rearrangements and the exposure of critical groups without altering the core folding pattern, thereby effectively modulating the functional properties of HbA. Moreover, we conducted a secondary structure detection, investigated the interaction mode between spermine and HbA, and performed conformational analysis on HbA. The impact of spermine on the secondary structure of HbA was determined through MMS. The HOS of HbA is primarily determined by intramolecular hydrogen bonds between amine hydrogen atoms and carbon–oxygen atoms in the peptide backbone. This structural arrangement plays a key role in determining the folding patterns of peptide units and the overall conformation of protein [50]. Our findings suggest that binding of spermine may lead to exposure of certain groups within the beta sheet region, causing partial α-helix in HbA to convert to β-sheet [51]. This conversion is accompanied by stable aggregation of intermolecular hydrogen bonds. The increase in oxygen affinity may be attributed to the above structural alteration [35]. However, the overall secondary structure of HbA remained largely unaffected. The similarity between HbA and HbA–spermine was found to be 98.2%. It elucidates why HbA retained the oxygen-carrying/releasing ability. Molecular docking analysis is a tool for predicting molecular binding affinity and activity [2]. In this study, we used molecular docking to investigate the binding site between spermine and HbA. The results revealed that spermine binds within the central hydrophobic cavity of HbA. It forms salt bridges with ASP99 (1.88 Å distance) of the β chain and GLU101 (1.93 Å distance) of the two β chains. Spermine also forms hydrogen bonds with THR38 (1.86 Å distance) and ASN97 (1.74 Å distance) of the α chain. The calculated CDOCKER-ENERGY value for this binding is 37.7944 kJ/mol. Notably, βAsp99 has been identified as a key amino acid site that reduces the oxygen affinity of HbA. It is in the switch region of the α1β2 dimer interface and forms a unique hydrogen bond interaction with α1Tyr42 and α1Asn97, stabilizing the “T” state [52]. Mutations in βAsp99, such as those found in Hb Ypsilanti (βAsp99Tyr), Hb Radcliffe (βAsp99Ala), and Hb Coimbra (βAsp99Glu), disrupt these hydrogen bond interactions, shift the allosteric equilibrium toward the “R” state, and increase HbA’s oxygen affinity [52]. Similarly, spermine binds βAsp99, thus increasing the oxygen affinity. Furthermore, βGLU101 has also been reported to regulate HbA’s oxygen affinity [2]. The interaction between spermine and βGLU101 also brings increased oxygen affinity of HbA, aligning with the theory for these amino acid sites.

The MDs were conducted to further determine the effect on HbA structure after binding spermine. The results revealed that spermine binds tightly to the central hydrophobic cavity of HbA. Throughout the 100 ns simulations, the protein–ligand complex remained structurally stable, as indicated by its lower RMSD values. The stability can be attributed to the presence of hydrogen bonds and van der Waals interactions within the complex. Salt bridge interactions were observed between spermine and Asp94, Asp239, Glu241, Glu525, and Asp527 of HbA. Hydrogen-bonding interactions were observed between spermine and Thr324 of HbA. The discrepancy in sites between the MD simulation and the molecular docking is attributed to their increasing stability throughout the simulation process. Upon the formation of these stable complexes, a notable conformational transition to the “R” state of HbA is also observed. It is evidenced by a significant decrease in the size of the β-cleft of HbA, primarily consisting of β1H146 and β2H146 [53]. Consequently, this conformational change results in an increased oxygen affinity of HbA. We used the MM/GBSA method to predict the binding free energy of the HbA–spermine complex as described by Khan et al. [54] by incorporating the polar solvation free energy. The calculated binding free energy, considering the contributions of non-polar solvation free energy and DGsolv fraction, depends on the solvation model utilized. Likewise, the DGgas fraction represents the van der Waals according to the molecular mechanics force field, coupled with the sum of electrostatic interactions independent of the solvation model used. The computed free energy value for the HbA–spermine complex was −26.0247 ± 10.0893 kcal/mol, suggesting its stability, as indicated by Irfan [55]. Therefore, both MDs and molecular docking suggest that spermine binds to key amino acid sites in the central water cavity of HbA and is responsible for regulating oxygen affinity. This binding mode leads to a stabilized HbA–spermine complex.

Our study reveals that the binding of spermine to HbA simultaneously boosts its antioxidant defense and confers a pH-responsive property. This finding directly tackles two persistent hurdles in HBOC development. Moving forward, we will explore two translational pathways rooted in the following properties: (i) harnessing the pH-dependent oxygen release for targeted delivery in hypoxic, acidic tissues—an elegant endogenous control strategy with broad relevance to HBOC design; and (ii) exploiting the improved stability to create a lyophilized, room-temperature-stable product essential for frontline and disaster medicine. Consequently, HbA–spermine emerges as a versatile platform for next-generation oxygen therapeutics, including portable resuscitative agents and enhanced organ preservation fluids.

## 4. Materials and Methods

### 4.1. Preparation of HbA, bHb, and Rat RBC

Volunteers’ blood was withdrawn through the median cubital vein. HbA was purified from whole blood mixed with CPDA-1 (Sigma Aldrich, St Louis, MO, USA) by anion-exchange chromatography and was prepared at a concentration of 5 g/dL, as previously described [56].

bHb was collected from *bovine* red blood cells. Fresh *bovine* whole blood was obtained from Beijing Ke Run Wei De Biotechnology (Beijing, China). bHb was purified according to a previously described method [57].

Healthy male Wistar rats (220–260 g; Vital River, Beijing, China) were anesthetized with sodium pentobarbital (Chinese Medicine Group Chemical Agent, Beijing, China, 50 mg/kg). Blood collection, storage, and leukoreduction were performed as described in the method [56].

### 4.2. Incubation of the HbA and Spermine

The HbA and spermine were incubated at pH 7.4. Spermine was dissolved in 100% dimethyl sulfoxide (PBS; Sigma Aldrich, St. Louis, MO, USA) to achieve a final concentration of 100 mM. HbA and spermine (molar ratio of 1:5, 1:25, 1:50, and 1:100, respectively) were uniformly mixed for 15 min. After incubation, final concentrations of HbA and spermine were 3 μM and 0.09–1.8 mM, respectively.

### 4.3. Thermal Denaturation Analysis and Static Light Scattering

The stability of HbA, both pre- and post-incubating with spermine, was assessed utilizing the All-in-One Biologics Stability Screening Platform (Unchained Labs, Norton, MA, USA). The purified HbA was diluted to a concentration of 1 mg/mL in PBS. Subsequently, the HbA solution was sampled with a HbA/spermine molar ratio of 1:50. The melting midpoint temperature (Tm1) was recorded as described in the method [58]. The first derivative curve of the barycentric mean (BCM) curve was computed and illustrated. The Tm1 and BCM of the maximum emission wavelength (Emax) were computed utilizing UNcle Analysis software (Unchained Labs, Norton, MA, USA, V.4.0). Static light scattering (SLS) data were concurrently gathered. The aggregation temperatures (Tagg266) were determined as the initial positive data point that exceeded the baseline of the first derivative of SLS. This first derivative was similarly computed using UNcle Analysis software.

### 4.4. Size and Polydispersity Index (PDI)

The sizes and PDIs of the HbA and HbA–spermine complex were evaluated by a dynamic light scattering (DLS) instrument (NanoBrook Omni, Brookhaven Instruments Corporation, Holtsville, NY, USA). The concentration of HbA was fixed at 5 µM, and the molar ratio of HbA to spermine was 1:50. The results were obtained by NanoBrook 21CFR Part-11 compliance software v. 3.5 [59].

### 4.5. Colloid Osmotic Pressure (COP)

The COP of HbA and HbA–spermine complex (molar ratio of 1:50) was measured by a Wescor 4420 Colloidal Osmometer (Wescor, Inc., Logan, UT, USA). Samples were dissolved in PBS buffer (pH 7.4), and the concentration of HbA was 7 mg/mL.

### 4.6. Oxygen Dissociation Assay (ODA)

ODA is a screening assay based on spectral changes in HbA during deoxygenation to represent the oxygen release of HbA [60]. HbA (3 µM) and HbA–spermine complex (molar ratio of 1:5, 1:25, 1:50, and 1:100) were oxygenated with air for 50 min (10 cycles) at 37 °C in 96-well optically transparent polystyrene plates. Then, deoxygenation was achieved by blowing N2 at 20 L/min in an ultraviolet/visible (UV/Vis) absorbance spectrometer (Omega, BMG Labtech, Inc., Ortenberg, Germany). During the process, spectral measurements (350–700 nm, with a spectral resolution of 1 nm) were obtained every 5 min as a cycle to determine the oxyHb level, and oxyHbA% is calculated as follows:

MetHb = 2.985 × (OD_630_ − OD_700_) +0.194 × (OD_576_ − OD_700_) − 0.4023 × (OD_560_ − OD_700_) (1)

Deoxy = 1.373 × (OD_560_ − OD_700_) − 0.747 × (OD_576_ − OD_700_) − 0.737 × (OD_630_ − OD_700_) (2)

OxyHb = 1.013 × (OD_576_ − OD_700_) − 0.3269 × (OD_630_ − OD_700_) − 0.7353 × (OD_560_ − D_700_) (3)

OxyHb% = Oxy-Hb/(Met-Hb + Deoxy + Oxy-Hb)(4)

The same method as described above was used to reveal the Bohr effect of HbA and the HbA–spermine complex. HbA and HbA–spermine (molar ratio of 1:50) were diluted in DPBS with pH values of 7.0, 7.2, 7.4, 7.6, and 8.0.

### 4.7. Antioxidant Experiments

The detection of MetHb was carried out in PBS buffer, pH = 7.4, at 37 °C. Absorbance changes in the range of 350–700 nm due to the spontaneous oxidation of Hbs were recorded using a UV-vis spectrometer at 1 h intervals for 8 h. A least-squares fitting method, as described in the literature, was employed to calculate MetHb concentrations by fitting the experimental spectra to known reference spectra [61,62]. The sample spectra are displayed in Figure 3A.

(5) $${D_{l}\left(\lambda \right)}_{theor}=\varepsilon_{HbO_{2},l}C_{{HbO}_{2}}L+\varepsilon_{Hb,l}C_{Hb}L+\varepsilon_{MetHb,l}C_{MetHb}L+K+S/\lambda^{4}$$

The model incorporates the following known parameters: the molar absorptivity coefficients at specific wavelengths λl($\varepsilon_{HbO_{2},l}$, $\varepsilon_{Hb,l}$, and $\varepsilon_{MetHb,l}$) and the optical path length L. The unknown parameters to be determined by the fitting procedure are the concentrations of the corresponding hemoglobin derivatives (CHbO_2_, CHb, and CMetHb) and the scattering coefficients K and S.

The capacity of the hydroxyl radical scavenging was ascertained utilizing the o-phenanthroline–ferrous iron chemistry method. The molar ratio of HbA to spermine was established at 1:50. Absorbances of the blank tube, control tube, and sample tube liquid at 536 nm were quantified as *A*0, *Ac*, and *At*, respectively. Clearance was then calculated using Formula (6) [63].

(6) $$\mathrm{Clearance}\;(\%)=\frac{At-Ac}{A0-Ac}\times 100\%$$

### 4.8. Oxygen Dissociation Curve (ODC)

HbA/HbA–spermine, bHb/bHb–spermine, and RBC/RBC–spermine were diluted in 4 mL of BLOODOX-Solution buffer with pH 7.4, mixed with 20 μL of *bovine* serum albumin (20%, Sigma Aldrich) and 20 μL of anti-foaming agent (Sigma Aldrich) [56]. Next, samples were analyzed using a BLOODOX-2018 analyzer (Softron Biotechnology, Beijing, China). The ODC and P50 values were recorded [56].

The same method as described above was used to reveal the Bohr effect of HbA and the HbA–spermine complex. HbA and HbA–spermine were diluted in BLOODOX-Solution buffer with pH values of 7.2, 7.4, and 7.6.

### 4.9. Surface Plasmon Resonance Technology (SPR)

The interaction between spermine (TargetMol, Wellesley Hills, MA, USA) and HbA at room temperature was determined using SPR instruments (Nicoya Life Science, Inc., Kitchener, ON, Canada) [35]. A carboxyl sensor chip was installed on the SPR instrument. HbA was diluted to 5 μg/μL with immobilization buffer (10 mM sodium acetate, pH 4.5). Spermine was diluted to 5, 2.5, 1.25, 0.625, and 0 μM with DMSO and injected at a flow rate of 20 μL/min for association for 240 s, followed by dissociation for 480 s. The association and dissociation processes were performed in the running buffer 1 × HEPES (10 mM HEPES, 150 mM NaCl, and 3 mM EDTA, with 0.005% Tween-20, pH 7.2, 7.4, and 7.6). Kinetic parameters of the binding reaction were calculated using Trace Drawer software V 1.10.1 (Ridgeview Instruments AB, Uppsala, Sweden) [64,65].

### 4.10. Microfluidic Modulated Spectroscopy (MMS)

Secondary structures of HbA and HbA–spermine complex were evaluated using MMS, which has been reported in the detection of Hb secondary structure [35]. Samples were analyzed using the AQS3^®^pro MMS production system (RedShiftBio, Burlington, MA, USA). The original absolute absorption spectra of samples were obtained in the range of 1701–1598 cm^−1^ and converted into the second derivative plot to highlight. Then the characteristic peak of the second-order structure was identified. Higher-order structure (HOS) components, alpha-helix (Alpha), beta-sheet (Beta), unordered, and beta-turn (Turn) were calculated. The analysis and spectral processing of samples were performed using the AQS3^®^ delta control software (RedShift BioAnalytics; accessed on 2024-09-10).

### 4.11. Molecular Docking

The computational analysis was conducted using Discovery Studio (version 2019). [66]. The 3D structures of HbA and spermine were downloaded from the Protein Data Bank [PDB ID: 1B86] and PubChem [CID: 1103], respectively. The position was optimized through the CHARMm force field after deleting water molecules and adding hydrogens. The prepared protein was considered as the target receptor for the CDOCKER protocol. Ten duplicates of Meriolin1 were generated and placed around the center of the set site. Each of these was subjected to molecular docking-based simulated annealing and final refinement by minimization, leading to 10 minimized docked poses. The ligand pose corresponding to the highest score was taken as the best-docked pose. The minimal binding free energy conformer was used for further analysis.

### 4.12. Molecular Dynamic Simulation (MD)

The molecular dynamics simulation of the Autodock vina V1.1.2 docking complex was performed using the AMBER 20 program. High-quality atomic charges of the ligand were generated by the AM1-BCC model. The solvation model used for molecular dynamics simulation was the optimal point charge model (OPC). A 10 Å truncated octahedron box has been used as the boundary conditions. The non-bonded cutoff is 10 Å. Systems were then minimized for 5000 energy steps while restraining the heavy atoms of HbA–spermine to relax hydrogens, water molecules, and salt ions. Then, 5000 steps of minimization were performed while restraining the HbA backbone. Restraints were removed, and the entire system was minimized for another 20,000 steps. The systems were thermalized from 0 to 300 K using the canonical NVT ensemble (number, volume, and temperature) at 1.0 atm for 20 ps [67]. The heated systems were then equilibrated at 300 K for 200 ps with restraint at 1.0 atm under isothermal–isobaric ensemble (NPT) conditions using a Langevin thermostat. Another 1 ns equilibration in the NVT system using the Berendsen thermostat. Production of 100 ns was then performed, and 5000 snapshots were generated for MM/GBSA calculations [68]. The MM/GBSA method was employed to calculate the binding energies averaged over 5000 snapshots in Amber 20. The PyMOL software V 2.3 was used for the illustration of the docked conformations. The 2D diagram was drawn by Discovery Studio 2019.

### 4.13. Statistical Analysis

All statistics are presented as mean ± standard deviation (SD). Statistical analysis was performed using SPSS 9.2 (IBM (Armonk, NY, USA), SPSS statistics 22). Plots displayed in the study represent the mean data from at least three experiments. Statistical analysis of data was performed with normality tests, and when the data do not meet the assumptions of normality and homoscedasticity, the Mann–Whitney U test is employed. One-way independent ANOVA was used for comparison between different groups. * *p* < 0.05 and ** *p* < 0.01 indicate statistical significance.

## 5. Conclusions

In summary, spermine interacts with βASP99, βGLU101, αTHR38, and αASN97 within the central cavity of HbA to form a stable complex, which induces a conformational shift in HbA to the “R” state and enhances oxygen transportation, along with a reduced autoxidation. Our findings highlight spermine as a valuable compound in the development of HBOCs.

## Figures and Tables

**Table 1:** The MM/GBSA Binding free energy (kcal/mol) for HbA–spermine complex.

	Generalized Born (GB)
ΔGvdw	−25.5808 ± 4.1208
ΔGele	−103.3406 ± 60.2447
ΔGpolar	106.1549 ± 54.2322
ΔGnonpolar	−3.2582 ± 0.3614
ΔGgas	−128.9214 ± 59.6415
ΔGsolv	102.8967 ± 54.2081
ΔGtotal	−26.0247 ± 10.0893

ΔGvdw, van der Waals energy; ΔGele, electrostatic energy; ΔGpolar, polar solvent effect; ΔGnonpolar, nonpolar solvation; ΔGgas, gas phase free energy; ΔGsol, solvation free energy.

## Data Availability

The original contributions presented in this study are included in the article. Further inquiries can be directed to the corresponding authors.

## Abbreviations

The following abbreviations are used in this manuscript:

HBOCs	Hemoglobin-based oxygen carriers
MetHb	Methemoglobin
Adult Hemoglobin	HbA
SOD	Superoxide dismutase
CAT	Catalase
PDA	Pyrroloquinoline quinone
Dex-Hb	Cross-linked hemoglobin
UV-Vis	Ultraviolet visible
RMSD	Root Mean Square Deviation
ROS	Reactive oxygen species
PDI	Polydispersity Index
DLS	Dynamic light scattering
COP	Colloid Osmotic Pressure
ODA	Oxygen Dissociation Assay
ODC	Oxygen Dissociation Curve
SPR	Surface Plasmon Resonance Technology
MMS	Microfluidic Modulated Spectroscopy
